# Musculus Palmaris Longus: Influence on Playing Capability of Keyboard Musicians – Preliminary Report

**DOI:** 10.3389/fpsyg.2018.01460

**Published:** 2018-08-13

**Authors:** Krzysztof Dąbrowski, Hanna Stankiewicz-Jóźwicka, Arkadiusz Kowalczyk, Michał Markuszewski, Bogdan Ciszek

**Affiliations:** ^1^Department of Descriptive and Clinical Anatomy, Medical University of Warsaw, Warsaw, Poland; ^2^Department of Piano, Harpsichord and Organ, The Fryderyk Chopin University of Music, Warsaw, Poland; ^3^Department of Choir Conducting, Music Education, Church Music, Rhythmics and Dance, The Fryderyk Chopin University of Music, Warsaw, Poland; ^4^Department of Neurosurgery in Bogdanowicz Children’s Hospital, Warsaw, Poland

**Keywords:** palmaris longus, wrist, hand, musician, keyboard, piano, strength, muscle

## Abstract

Musculus Palmaris Longus (PL) is one of the most variable anatomical structures in the human body. Despite being biomechanically active, it is vastly considered to have no impact on the functionality of the upper extremity in the general population. The aim of this study is to examine the relation between playing capability of young musicians and morphology of Musculus PL and to compare it with the relation between manual capability of non-musicians and morphology of their Musculus PL. 42 forearms of 21 healthy individuals (11 musicians and 10 non-musicians) were subjected to Shaeffer’s test and ultrasound imaging and tested by dynamometer for hand grip strength and the first and fifth finger opposition before and after exertion. No difference in morphology pattern was observed between the groups. In the musicians, a substantial loss of a hand grip strength of the left hand compared to the right hand after exertion, regardless of lateralization, was observed. A disproportion in exhaustion of the musician’s hands with unilateral absence of PL was observed – the difference in grip strength between the dominant and non-dominant hand before and after exertion increased over eight times more than in the musicians with bilateral presence. There is no difference in PL morphology between either the musicians or non-musicians. Regardless of lateralization, the musician’s left hand in musicians seems weaker and therefore more prone to misuse related injuries. PL may play a role in musicians in balancing muscular exhaustion.

## Introduction

Musical activity constitutes a great challenge for both amateur and professional musicians, especially the one involving classical instruments such as a grand piano. According to the research conducted by Leaver et al. (2011), 86% of professional orchestra musicians confess suffering from pain in the past 12 months and 41% would describe the pain as “disabling.” Moreover, 86% of musicians report that their musical training routine involves over 4 h of repeated fingers and wrist movements each day.

Such problems seem to have been known for over a century – Poore described muscular overuse in pianists in 1887 and compared it to a writer’s cramp – and appear to be present from early stages of musical training, with half of the students admitting musculoskeletal pain and locating it in their wrists and hands, and with vast majority of them suffering loss in playing technique (Fry, 1987). Analysis of the muscular system in a musician’s forearm is a rare topic in scientific literature. Therefore, we started our investigation with Musculus Palmaris Longus (PL), it being both easily accessible and generally neglected structure.

**FIGURE 1:**
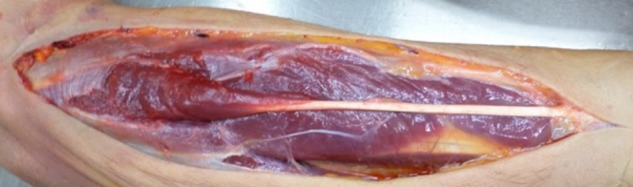
Musculus Palmaris Longus on fresh cadaver.

Musculus PL is usually described as a long, slender, spindle-shaped muscle with its proximal insertion at medial epicondyle of the humerus and distal insertion in palmar aponeurosis (Agarwal, 2010) (**Figure 1**). It is believed to be an atavistic structure that used to enhance grip in primeval apes. In humans, it is considered to be one of the most variable muscles, with most common variation being its absence – which has proven to be related primarily to the ethnicity of the examined individuals (Thompson et al., 2001; Deniz and Yldiz, 2011; Barkats, 2014b; Roqueline et al., 2014). PL is classified as a weak wrist flexor and, through the palmar aponeurosis, flexor of the metacarpophalangeal joints. Moreover, it seems to be most selectively engaged in the opposition of the first and fifth fingers. There is some research linking certain variations of the muscle with hand and wrist dysfunctions – for example, Musculus PL Invertus (variation with the belly of the muscle lying in distal, instead of typical proximal part of the forearm) seems to be significant risk factor in Carpal Tunnel Syndrome. Despite all that, PL is widely considered redundant as either its absence or presence appears to have no impact on the upper extremity function. Its significance, according to the current medical knowledge, seems to be limited to being a convenient material in tendon grafts and other reconstructive procedures such as ptosis correction or glans penis coronaplasty, genetics and antropomorphics studies, or as a landmark in radiology or surgical procedures (Agarwal, 2010; Deniz and Yldiz, 2011; Kigera and Mukwaya, 2011; Syaiful and Dody, 2015).

Action of PL by palmar aponeurosis may affect possibility of closing hand or the force of maintaining partially flexed fingers, which seems to be characteristic for any activity related to the use of a keyboard. Of course, the use of a computer keyboard or an electronic instrument keyboard requires much less intensive effort than the use of a classic grand piano keyboard. Therefore, the aim of this study is to examine and describe influence of PL’s variability, especially its absence, on playing capability of keyboard musicians and to compare it to its influence on the manual capability of the people without the history of advanced musical training.

## Materials and Methods

Materials consisted of 42 hands and forearms of 21 healthy individuals. All the participants signed the form of informed consent and had opportunity to ask any questions they deemed relevant. The group of 11 keyboard musicians (aged 19–38) had their PL’s morphology examined first by Schaeffer’s test and then by an ultrasound imaging. Their hand grip strength and selectively the first and fifth finger opposition strength was measured by digital dynamometer (KERN MAP 80K1S) before and after a 15 min piano playing test designed especially for the sake of this study. The piano playing test consisted of continuous playing of scales and passages with both hands simultaneously. The scales and passages were chosen to remain within the comfort zone of the particular participant and speed and the dynamics of playing were altered by the observing instrument teacher during the test to reach the similar level of exhaustion in all the participants regardless of their skill and physical prowess. The instrument teacher and the medical professional were present and both verbal and visual contact with the participant were maintained to ensure reliable results while avoiding any unnecessary risks. The results were compared to the results of non-musician control group (10 people, aged 20–25) with the piano playing test being substituted with a 15 min hand grip stamina test consisting of deforming a gel ball (of initial pressure resistance similar to that of the key pressure resistance of the grand piano in use) with fingertips in a specific way. The comparison involved multiple angles of analysis including mean differences in dominant and non-dominant hand before and after exertion as well as tests for statistical significance.

The entire procedure was completely non-invasive and the precautions were taken to avoid any negative impact on the participants health and well-being.

The ultrasound machine used during the examination was LOGIQ F8 GE with L6-12, 6–13 MHz probe.

## Results

Among the musicians examined, one person with unilateral absence of PL (left, non-dominant) was found as well as one person with unilateral absence (right, non-dominant) and only residual muscle (reduced to a tendon connecting palmar aponeurosis with musculus flexor digitorum superficialis) in the other hand (left, dominant).

In the control group there were recognized: one person with bilateral absence of PL, one person with unilateral absence (left, non-dominant), one person with bilateral presence with one muscle’s proximal insertion being aponeurosis antebrachii (right, dominant), one person with bilateral presence with one muscle (right, dominant) being inverted and one person with bilateral presence, whose muscle’s tendon ramified in the muscle’s belly in both hands. Those variations are categorized as quality variations and have been observed and described before (Ciszek and Szaro, 2003; Pires et al., 2017).

In both groups, there were no significant differences in the results of the dynamometer tests between specimen with bilateral presence, bilateral absence or unilateral absence of PL (**Tables 1, 2**).

**Table 1:** Selected strength measurements for the group of musicians.

Number	1	2	3	4	5	6	7	8	9	10	11
Sex	F	F	M	M	F	F	M	M	M	F	F
Dominant	R	R	L	R	L	R	R	R	L	R	R
Presence	Yes/yes	Yes/yes	Yes/yes	No/yes	Tendon/no	Yes/yes	Yes/yes	Yes/yes	Yes/yes	Yes/yes	Yes/yes
I grip test before (kgf)	27/27.5	26/27.7	34.4/31.6	41.4/43.2	44.4/35.2	27.2/33.1	32.3/34.8	44.2/43.3	44.5/39.1	29.5/29.9	22.3/28.1
II grip test before (kgf)	24.6/27.3	26.3/25.6	38.1/33.1	39.4/43.2	38.1/34.8	25.9/30	32.1/32.5	40.8/43.2	41.5/38.5	26.7/26.5	20.3/22.9
III grip test before (kgf)	21.8/26.6	26.6/23.7	33.3/32.7	37.3/42.6	39.7/37.1	25.5/28.7	31.3/39.8	44.4/43.3	42.6/37.7	24.7/26.5	20.7/21.4
Mean (kgf)	24.5/27.2	26.3/25.7	35.3/32.8	39.4/43	40.7/35.7	26.2/30.6	31.9/35.7	43.1/43.3	42.9/38.4	27/27.6	21.1/24.1
I grip test after (kgf)	23.8/31.1	26.8/27.9	22.6/26	41/48.1	39.5/36.6	26.7/32.1	32/38	43.1/44.5	41.8/40.6	31.2/29.3	16.2/29.4
II grip test after (kgf)	20.1/28.8	24.9/24	23.7/23.2	36.8/43	38.2/33.5	25.8/30.8	27.8/35	42.8/44.5	39.7/40.7	28.4/27.4	17.8/20.3
III grip test after (kgf)	21.8/27.8	21.5/20.3	22.8/23	35.1/45.2	35/35.2	26.8/31.2	31.5/36.2	38.9/44.7	38.3/40.3	27.8/27	16.8/21.7
Mean (kgf)	21.9/29.2	24.4/24.1	23/24	37.6/45.4	37.6/35.1	26.4/31.4	30.4/36.4	41.6/44.6	39.9/40.5	29.1/27.9	16.9/23.8
I opp. test before (kgf)	5.0/3.6	3.7/4.6	2.7/2.4	3.7/5.2	5.3/4.9	2.3/2.2	5.3/5.9	3.7/4.6	2.9/3.4	4.5/4.7	4.2/5.0
II opp. test before (kgf)	4.3/3.5	2.9/3.3	2.4/2.2	4.0/4.2	5.4/4.9	2.6/2.3	5.6/6	2.7/3.2	3.1/3.4	4.4/4.6	4.1/4.4
III opp. test before (kgf)	4.5/3.0	3.2/2.7	2.6/2.3	4.0/4.7	5.2/4.7	3.1/3.3	5.1/5.4	2.4/4.1	3.0/3.4	4.4/4.4	3.7/4.3
Mean (kgf)	4.6/3.4	3.3/3.5	2.6/2.3	3.9/4.7	5.3/4.8	2.7/2.6	5.3/5.8	2.9/4.0	3.0/3.4	4.4/4.6	4/4.6
I opp. test after (kgf)	3.5/3.1	3.7/3.8	2.3/2.3	5.1/6.2	4.8/4.7	2.3/2.2	5.7/5.8	2.5/3.4	2.5/2.2	3.5/5.0	3.5/4.4
II opp. test after (kgf)	3.7/3.2	3.4/3.1	2.2/2.5	5.4/4.3	4.9/4.1	2.2/2.1	5.1/6	2.7/3.0	2.3/2.3	4.2/4.7	3.6/4.1
III opp. test after (kgf)	3.7/3.4	3.3/3.8	2.1/2.3	5.4/4.3	5.0/2.4	2.6/2.3	5.3/5.7	2.2/3.1	2.3/2.1	3.8/4.0	3.3/4.1
Mean (kgf)	3.6/3.2	3.5/3.6	2.2/2.4	5.3/4.9	4.9/3.7	2.4/2.2	5.4/5.8	2.5/3.2	2.4/2.2	3.8/4.6	3.5/4.2

**Table 2:** Selected strength measurements for the group of non-musicians.

Number	1	2	3	4	5	6	7	8	9	10
Sex	M	F	F	M	F	M	F	M	F	F
Dominant	R	R	R	R	R	R	R	R	R	L
Presence	Yes/yes	Yes/yes	Yes/yes	No/no	Yes/flexor	No/yes	Yes/yes	Yes/invert.	Yes/Yes	Yes(r)/yes(r)
I grip test before (kgf)	47.6/52.9	34.2/34.7	21.3/30.2	45.5/43.4	28.9/31.1	27.8/37.3	24.2/27.4	45.6/49.1	27/32.2	26.1/24.7
II grip test before (kgf)	45.7/47.7	33.4/36.2	23.6/29.7	43/42.2	28.7/31.6	28.9/34.2	20.9/22.1	44.6/43.3	27.5/32.4	25.4/25.8
III grip test before (kgf)	42/46	31/34.2	20.1/24.3	45.4/43.7	26.9/33.1	26.9/33.4	21.1/25.9	41.9/43.6	24.9/31.3	25.5/25.4
Mean (kgf)	45.1/48.9	32.9/35	21.7/28.1	44.6/43.1	28.2/31.9	27.9/35	22.1/25.1	44/45.3	26.5/32	25.7/25.3
I grip test after (kgf)	37.9/40.6	31.5/36.9	25.3/27.9	43.2/39.9	31.8/33.8	25.8/36.5	16.5/27.4	42.4/46.3	31.4/30.3	24.1/25.8
II grip test after (kgf)	38.2/38.9	28.8/34.5	23.1/24.9	42.8/42.2	29.7/33.9	24.2/28.3	23/24.9	42.8/41.2	27.9/29.9	22.1/20.7
III grip test after (kgf)	31.8/38	28.1/32.2	21.2/23.2	38.7/41.8	29.1/33.4	25.9/32	18/23.7	39.8/39.9	25.7/31.7	23.8/23.3
Mean (kgf)	36/39.2	29.5/34.5	23.2/25.3	41.6/41.3	30.2/33.7	25.3/32.3	19.2/25.3	41.7/42.5	28.3/30.6	23.3/23.3
I opp. test before (kgf)	4.3/4.9	2.3/2.2	3.4/2.8	4.3/6.3	3.3/4.1	3.0/4.4	2.3/2.4	4.7/4.5	4.0/2.3	2.2/2.1
II opp. test before (kgf)	4.0/5.0	2.5/2.2	3.3/2.4	3.4/6.5	3.0/3.6	3.0/2.1	3.5/2.8	3.8/3.6	3.7/2.6	2.5/3.3
III opp. test before (kgf)	4.0/3.4	2.3/2.4	3.3/2.9	4.4/6.4	2.8/3.6	2.5/3.6	2.1/2.6	4.1/2.7	4.8/2.6	2.3/2.4
Mean (kgf)	4.1/4.4	2.4/2.3	3.3/2.7	4.0/6.4	3.0/3.8	2.8/3.4	2.6/2.6	4.2/3.6	4.2/2.5	2.3/2.6
I opp. test after (kgf)	3.7/6.7	2.3/2.5	3.3/2.8	4.3/6.1	2.5/4.1	2.9/4.1	2.2/2.5	3.4/5.1	4.0/3.1	2.7/2.2
II opp. test after (kgf)	4.9/6.0	2.4/2.8	3.1/2.6	4.4/6.2	2.9/3.4	3.0/3.5	2.1/2.3	2.3/3.9	4.3/2.9	2.4/2.7
III opp. test after (kgf)	4.5/6.6	2.4/2.6	3.3/2.7	4.6/6.1	2.3/3.4	2.2/5.2	2.1/2.4	2.4/3.4	2.8/3.1	2.3/2.8
Mean (kgf)	4.4/6.4	2.4/2.6	3.2/2.7	4.4/6.1	2.6/3.6	2.7/4.3	2.1/2.4	2.7/4.1	3.7/3.0	2.5/2.6

Among the musicians, there was a noticeable difference in the mean of grip strength loss between the left and right hand after the exertion (2.8 kgf loss in the left hand and 0.4 kgf loss in the right hand) regardless of lateralization. The tendency was tested for statistical significance by the paired Student’s test with *p*-value for the left hand difference being *p* = 0.018 and *p*-value for the right hand being *p* = 0.69. Such clear tendency did not occur in the control group (1.8 kgf loss in the left hand and 2.2 kgf loss in the right one) with *p*-value for the left hand being *p* = 0.08 and *p*-value for the right hand being *p* = 0.05.

Most noticeable tendency was observed in changes of mean difference between the dominant and non-dominant hand before and after exertion, in the context of presence or absence of PL. In the case of the musician with unilateral absence of PL, the difference in the grip strength and opposition strength after exertion increased by 4.2 and 1.2 kgf. In the group of the musicians with both muscles present, the mean difference in strength increased only by 0.5 and 0.1 kgf. Such tendency was not observed in the control group (analogically – increase of 0.4 and 0.7 kgf for unilateral presence and 0.1 and 1 kgf for bilateral presence). However, this observation was not supported by Grubbs’ test for outliners.

## Discussion

The dynamometer tests before and after the exertion allowed a little insight regarding the kind of toll that playing a keyboard instrument takes on upper extremities. Initially, there seemed to be no significant difference in strength between the groups of musicians and non-musicians or between the participants with bilateral absence, unilateral absence or bilateral presence of PL. However, after closer inspection a promising observation was made. After 15 min of playing the instrument with both hands simultaneously and at the same intensity the musicians lost on average 2.8 kgf (kilogram-force) of hand-grip strength in their left hands and only 0.4 kgf in their right hands. Testing the outcome with the paired Student’s test for statistical significance showed the *p*-value for the left hand to be *p* = 0.018 and the *p*-value for the right hand to be *p* = 0.69. What seems even more interesting, the disproportion of the strength loss did not variate in the case of three musicians classified as left-hand dominant even though their original left hand-grip strength was noticeably bigger (*p*-values for dominant and non-dominant hands being *p* = 0.275 and *p* = 0.052). Such disproportion did not occur in the control group which scored on average 1.8 kgf loss in the left hand (*p* = 0.08) and 2.2 kgf loss in the right one (*p* = 0.05). The testing of the first and fifth finger opposition showed 0.1 kgf (left) and 0.3 kgf (right) strength loss in the musicians and 0.2 kgf (left) strength loss and 0.4 kgf (right) strength gain in the control group.

The substitute stamina test for the control group, although the activity in question may appear very different, seems legitimate enough. It is hard to imagine that a person without any musical training is able to perform even the simplest exercise on the keyboard of the grand piano for 15 min without stopping, while it could be speculated that squeezing a gel ball with fingertips should be an activity just as natural – and yet demanding some attention – as simple exercises on the keyboard are for someone with many years of training. Moreover, the control group participants were instructed to use all the fingers with equal strength and to work the ball at specific frequency, since it has been proven that for a person without musical training even individual keystrokes cause co-activation of the uninvolved fingers, and rhythmical, faster keystrokes are much more engaging for the forearm muscles (Chong et al., 2015). The control group test was aimed to incorporate those factors as well.

**FIGURE 2:**
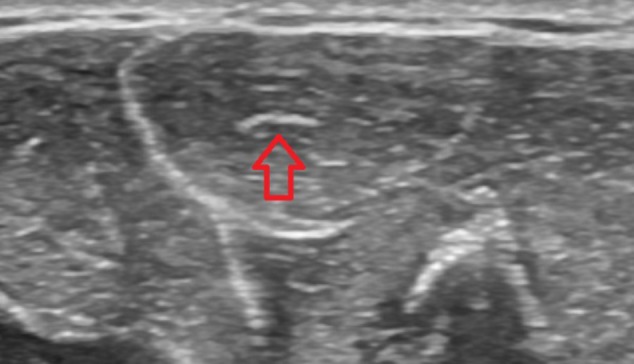
Ultrasound imaging of spindle-shaped PL with a visible tendon inside of the muscle’s belly (red arrow).

There seems to be no significant difference in PL belly’s morphology between the musicians and non-musicians. Shaeffer’s test, used vastly in both research and clinic due to its simplicity along other clinical tests such as Gangata, Mishra’s, Thompson’s, or Pushpakumar’s (Agarwal, 2010; Deniz and Yldiz, 2011; Kigera and Mukwaya, 2011; Hussain and Hasan, 2012; Syaiful and Dody, 2015) gave two false-negative results in both groups, when compared to ultrasound imaging. The frequency of absence of PL was at 15% in both groups, in a pattern much closer to general European-Caucasian population (15.2%) than strictly Polish population (8.6%) (Hussain and Hasan, 2012). The proportion of pennated and bipennated muscle bellies and spindle-shaped muscle bellies is the same as well. Moreover, such classification could be questioned since all the muscle bellies presenting themselves as spindle-shaped in the ultrasound imaging proved to have a tendon within the belly, which contradicts the definition of spindle-shaped muscle in the first place (Dąbrowski et al., 2017) (**Figure 2**). Both groups defy Loth’s description of Caucasian PL which includes the length of PL’s tendon dominating over the length of its belly (Loth, 1931). This could not be considered a local anomaly such as in Ukrainian village of Dercen, where strictly Hungarian origin of the citizens has created a unique pattern of PL agenesis (70% of population born before 1945) (Barkats, 2014a), since this study included students hailing from various sites of Poland. The differences between the groups in that matter appear only in singular cases such as single musculus PL invertus found in the control group. Limited amount of such cases prevents any speculations regarding a potential pattern.

Extended analysis of the dynamometer test results revealed an interesting pattern. The difference of inequality in strength between the dominant and non-dominant hand before and after exertion noticeably escalated in the case of the musician with unilaterally absent PL. In their case the grip strength difference between the dominant and non-dominant hand increased after exertion from 3.6 to 7.8 kgf, which marks a change of 4.2 kgf and the opposition strength difference changed from 0.8 to –0.4 kgf, which marks a change of 1.2 kgf. In the case of the musicians with bilateral presence of PL the mean of total change of differences showed 0.5 kgf in hand grip and 0.1 kgf in opposition, which in comparison seems very consistent. These results were statistically analyzed and proved to have normal distribution. However, Grubbs’ test showed no statistically significant outliners in the group (*P* > 0.05) which means that observed difference changes could fall within the norm and the examination of more musicians with PL’s unilateral absence is required. In the control group, the person with unilateral absence of PL showed a total change of differences equal to 0.1 kgf change in hand grip and 1 kgf change in opposition. The control group members with bilateral PL presence indicated a change of differences of 0.4 kgf in hand grip and 0.7 kgf in opposition. The results of the control group member with unilateral absence do not follow the pattern of the musician with unilateral absence, however, the results of the musicians with bilateral presence and non-musicians with bilateral presence seem to be more similar, especially regarding the hand grip strength. It could be speculated that the unilateral absence of PL may be responsible for the inconsistent changes in strength differences between the dominant and non-dominant hand of the keyboard musician, which in return could mean that PL may play a certain role in evening out the hand and forearm exhaustion while playing a keyboard instrument such as the grand piano. To either support or debunk this hypothesis, an examination of a bigger group is in order.

## Conclusion

The tests designed specifically to put the same load on both hands of the musicians have shown a big difference in grip strength loss between the left and right hand, regardless lateralization (3 people noted as left-dominant) which may indicate that the left hand is on average the weaker one and therefore more prone to misuse related injuries.

Moreover, the changes in differences between the dominant and non-dominant hand before and after exertion seem to suggest that presence of Musculus PL may result in more evenly balanced muscle exhaustion specifically among keyboard musicians.

Due to complex relations between variables and limited amount of specimen, use of statistical significance tests in this stage seems not valuable, therefore the study will be continued and expanded.

## Ethics Statement

This study was exempt from approval by Bioethics Commitee of Medical University of Warsaw due to its complete non-invasiveness and its pilot nature. All subjects have signed a form of informed consent in accordance with the Declaration of Helsinki. All subjects have joined willingly with full knowledge of the requirements and procedures of the study and knowledge that they could leave the study at any time without giving any reasons and without any repercussions.

## Author Contributions

KD, HS-J, and BC contributed to the study conception and the design. HS-J, AK, and MM contributed to the gathering of test subjects. KD, HS-J, AK, and MM contributed to the data acquisition. KD contributed to data analysis and writing of the paper.

## Conflict of Interest Statement

The authors declare that the research was conducted in the absence of any commercial or financial relationships that could be construed as a potential conflict of interest.
